# Supplementary material for: Residential neighbourhood greenspace is associated with reduced risk of cardiovascular disease: A prospective cohort study
Source: PLoS One. 2020 Jan 3;15(1):e0226524. doi: 10.1371/journal.pone.0226524 (PMC6941810; doi:10.1371/journal.pone.0226524)
Supplement: S1 Table — (DOCX) [file pone.0226524.s001.docx]

**S1 Table: Sensitivity testing**

Hazard ratios from Cox regression, showing the association between, and total, direct and indirect (via physical activity) effects of, neighbourhood greenspace exposure and incident CVD, according to different definitions of neighbourhood and exposure.

*Notes:* *Age is used as the underlying time scale, n=24420. Model 2 adjusted for confounders of sex, age, BMI, prevalent diabetes and SES (individual and neighbourhood) (n=23759). CI: confidence interval.*

| 800m circular buffer | Model 1  Adjusted for greenspace | | | | |  | Model 2  Adjusted for confounders | | | | |
| --- | --- | --- | --- | --- | --- | --- | --- | --- | --- | --- | --- |
|  |  | 95% CI | |  |  |  |  | 95% CI | |  |  |
|  | HR | Lower | Upper | p | p trend |  | HR | Lower | Upper | p | p trend |
| Greenspace quartile  1 (least green, ref) | 1.00 |  |  |  | <0.001 |  | 1.00 |  |  |  | 0.001 |
| 2 | 0.96 | 0.92 | 1.01 | 0.136 |  |  | 0.97 | 0.92 | 1.02 | 0.189 |  |
| 3 | 0.92 | 0.88 | 0.96 | 0.001 |  |  | 0.92 | 0.88 | 0.97 | 0.002 |  |
| 4 (most green) | 0.92 | 0.88 | 0.97 | 0.001 |  |  | 0.93 | 0.88 | 0.97 | 0.003 |  |
|  |  |  |  |  |  |  |  |  |  |  |  |
| Effect (least versus most green quartile) |  |  |  |  |  |  |  |  |  |  |  |
| Total effect | 0.92 | 0.88 | 0.97 | 0.001 |  |  | 0.93 | 0.88 | 0.97 | 0.003 |  |
| Direct effect | 0.93 | 0.91 | 0.96 | <0.001 |  |  | 0.94 | 0.91 | 0.96 | <0.001 |  |
| Indirect effect (via physical activity) | 0.99 | 0.97 | 1.01 | 0.437 |  |  | 0.99 | 0.97 | 1.01 | 0.416 |  |

| 800m road buffer | Model 1  Adjusted for greenspace | | | | |  | Model 2  Adjusted for confounders | | | | |
| --- | --- | --- | --- | --- | --- | --- | --- | --- | --- | --- | --- |
|  |  | 95% CI | |  |  |  |  | 95% CI | |  |  |
|  | HR | Lower | Upper | p | p trend |  | HR | Lower | Upper | p | p trend |
| Greenspace quartile  1 (least green, ref) | 1.00 |  |  |  | 0.001 |  | 1.00 |  |  |  | 0.002 |
| 2 | 1.00 | 0.95 | 1.04 | 0.867 |  |  | 0.98 | 0.94 | 1.03 | 0.511 |  |
| 3 | 0.92 | 0.88 | 0.97 | 0.001 |  |  | 0.92 | 0.88 | 0.97 | 0.002 |  |
| 4 (most green) | 0.94 | 0.89 | 0.99 | 0.011 |  |  | 0.94 | 0.89 | 0.99 | 0.012 |  |
|  |  |  |  |  |  |  |  |  |  |  |  |
| Effect (least versus most green quartile) |  |  |  |  |  |  |  |  |  |  |  |
| Total effect | 0.94 | 0.89 | 0.99 | 0.011 |  |  | 0.94 | 0.89 | 0.99 | 0.012 |  |
| Direct effect | 0.95 | 0.93 | 0.97 | <0.001 |  |  | 0.95 | 0.92 | 0.97 | <0.001 |  |
| Indirect effect (via physical activity) | 0.99 | 0.97 | 1.02 | 0.465 |  |  | 0.99 | 0.97 | 1.01 | 0.429 |  |

| 3km circular buffer | Model 1  Adjusted for greenspace | | | | |  | Model 2  Adjusted for confounders | | | | |
| --- | --- | --- | --- | --- | --- | --- | --- | --- | --- | --- | --- |
|  |  | 95% CI | |  |  |  |  | 95% CI | |  |  |
|  | HR | Lower | Upper | p | p trend |  | HR | Lower | Upper | p | p trend |
| Greenspace quartile  1 (least green, ref) | 1.00 |  |  |  | 0.016 |  | 1.00 |  |  |  | 0.011 |
| 2 | 0.89 | 0.85 | 0.93 | <0.001 |  |  | 0.91 | 0.86 | 0.96 | <0.001 |  |
| 3 | 0.94 | 0.90 | 0.99 | 0.010 |  |  | 0.93 | 0.88 | 0.97 | 0.003 |  |
| 4 (most green) | 0.92 | 0.88 | 0.97 | 0.001 |  |  | 0.92 | 0.88 | 0.97 | 0.002 |  |
|  |  |  |  |  |  |  |  |  |  |  |  |
| Effect (least versus most green quartile) |  |  |  |  |  |  |  |  |  |  |  |
| Total effect | 0.92 | 0.88 | 0.97 | 0.001 |  |  | 0.92 | 0.88 | 0.97 | 0.002 |  |
| Direct effect | 0.93 | 0.91 | 0.95 | <0.001 |  |  | 0.93 | 0.90 | 0.95 | <0.001 |  |
| Indirect effect (via physical activity) | 0.99 | 0.97 | 1.02 | 0.576 |  |  | 0.99 | 0.97 | 1.02 | 0.583 |  |

| 3km road buffer | Model 1  Adjusted for greenspace | | | | |  | Model 2  Adjusted for confounders | | | | |
| --- | --- | --- | --- | --- | --- | --- | --- | --- | --- | --- | --- |
|  |  | 95% CI | |  |  |  |  | 95% CI | |  |  |
|  | HR | Lower | Upper | p | p trend |  | HR | Lower | Upper | p | p trend |
| Greenspace quartile  1 (least green, ref) | 1.00 |  |  |  | 0.003 |  | 1.00 |  |  |  | 0.001 |
| 2 | 0.93 | 0.89 | 0.97 | 0.002 |  |  | 0.94 | 0.90 | 0.99 | 0.021 |  |
| 3 | 0.93 | 0.89 | 0.98 | 0.003 |  |  | 0.92 | 0.88 | 0.97 | 0.002 |  |
| 4 (most green) | 0.92 | 0.88 | 0.97 | 0.002 |  |  | 0.92 | 0.87 | 0.97 | 0.002 |  |
|  |  |  |  |  |  |  |  |  |  |  |  |
| Effect (least versus most green quartile) |  |  |  |  |  |  |  |  |  |  |  |
| Total effect | 0.92 | 0.88 | 0.97 | 0.002 |  |  | 0.92 | 0.87 | 0.97 | 0.002 |  |
| Direct effect | 0.93 | 0.91 | 0.96 | <0.001 |  |  | 0.93 | 0.90 | 0.95 | <0.001 |  |
| Indirect effect (via physical activity) | 0.99 | 0.97 | 1.02 | 0.530 |  |  | 0.99 | 0.97 | 1.02 | 0.564 |  |

| 5km circular buffer | Model 1  Adjusted for greenspace | | | | |  | Model 2  Adjusted for confounders | | | | |
| --- | --- | --- | --- | --- | --- | --- | --- | --- | --- | --- | --- |
|  |  | 95% CI | |  |  |  |  | 95% CI | |  |  |
|  | HR | Lower | Upper | p | p trend |  | HR | Lower | Upper | p | p trend |
| Greenspace quartile  1 (least green, ref) | 1.00 |  |  |  | <0.001 |  | 1.00 |  |  |  | <0.001 |
| 2 | 0.92 | 0.87 | 0.96 | <0.001 |  |  | 0.95 | 0.90 | 1.00 | 0.040 |  |
| 3 | 0.92 | 0.88 | 0.96 | <0.001 |  |  | 0.91 | 0.87 | 0.96 | <0.001 |  |
| 4 (most green) | 0.91 | 0.86 | 0.95 | <0.001 |  |  | 0.91 | 0.86 | 0.96 | <0.001 |  |
|  |  |  |  |  |  |  |  |  |  |  |  |
| Effect (least versus most green quartile) |  |  |  |  |  |  |  |  |  |  |  |
| Total effect | 0.91 | 0.86 | 0.95 | <0.001 |  |  | 0.91 | 0.86 | 0.96 | <0.001 |  |
| Direct effect | 0.92 | 0.89 | 0.94 | <0.001 |  |  | 0.92 | 0.89 | 0.94 | <0.001 |  |
| Indirect effect (via physical activity) | 0.99 | 0.97 | 1.02 | 0.515 |  |  | 0.99 | 0.97 | 1.02 | 0.556 |  |

| 5km road buffer | Model 1  Adjusted for greenspace | | | | |  | Model 2  Adjusted for confounders | | | | |
| --- | --- | --- | --- | --- | --- | --- | --- | --- | --- | --- | --- |
|  |  | 95% CI | |  |  |  |  | 95% CI | |  |  |
|  | HR | Lower | Upper | p | p trend |  | HR | Lower | Upper | p | p trend |
| Greenspace quartile  1 (least green, ref) | 1.00 |  |  |  | <0.001 |  | 1.00 |  |  |  | <0.001 |
| 2 | 0.92 | 0.87 | 0.96 | <0.001 |  |  | 0.95 | 0.90 | 1.00 | 0.045 |  |
| 3 | 0.95 | 0.90 | 0.99 | 0.020 |  |  | 0.93 | 0.89 | 0.98 | 0.008 |  |
| 4 (most green) | 0.90 | 0.86 | 0.94 | <0.001 |  |  | 0.91 | 0.86 | 0.95 | <0.001 |  |
|  |  |  |  |  |  |  |  |  |  |  |  |
| Effect (least versus most green quartile) |  |  |  |  |  |  |  |  |  |  |  |
| Total effect | 0.90 | 0.86 | 0.94 | <0.001 |  |  | 0.91 | 0.86 | 0.95 | <0.001 |  |
| Direct effect | 0.91 | 0.89 | 0.93 | <0.001 |  |  | 0.91 | 0.89 | 0.94 | <0.001 |  |
| Indirect effect (via physical activity) | 0.99 | 0.97 | 1.02 | 0.490 |  |  | 0.99 | 0.97 | 1.02 | 0.533 |  |
