# Supplementary material for: Residential neighbourhood greenspace is associated with reduced risk of cardiovascular disease: A prospective cohort study
Source: PLoS One. 2020 Jan 3;15(1):e0226524. doi: 10.1371/journal.pone.0226524 (PMC6941810; doi:10.1371/journal.pone.0226524)
Supplement: Attachment — Submitted filename: ResponseToReviewers.docx [file pone.0226524.s002.docx]

**Editor comments**

Comment 1

Major comments are as follows;

1) Authors reported that they found effect modifications by age, sex, and occupations. Please present p-values for effect modification by age, sex, and occupations. It does not seem to be clear whether there was a significant interaction by these potential interactions. for example, regarding strata by age (N = 5325) vs. (N = 17409), sample size matters for a significant association.

*Response 1*

*Thank you for raising this issue. When investigating this, we discovered an error in the data, whereby Stata had incorrectly coded missing values as 1 for the manual occupation variable (n=133). As a result, all regression models that had been adjusted for covariates needed to be re-run, including the models for the sensitivity analysis. Tables 3 and 4 in the manuscript, as well as and the supplementary file, have been updated accordingly. The findings and conclusions of the paper remain substantively unchanged, and the statistical significance as the direction of association were unaffected. The median analysis was unaffected as it was conducted in R, which correctly coded the missing data.*

*Reanalysis found no significant interactions between greenspace and age or sex. Models 5, 6, 7 and 8 have now been removed from Table 4. The abstract and discussion now just state that occupation moderated the relationship (L33-4 and L281-4). We explicitly now state in the results ‘There was no statistically significant interaction between greenspace and age or sex in the regression models’ (L254-5).*

Comment 2

2) Please include person-years and no. of cases for each quartile for Tables 3 and 4. (for a dose-response meta-analysis in the future)

*Response 2*

*This has now been added to the tables.*

Comment 3

There are some minor comments.

1) Please correct the following sentence;

Abstract:

Socio-economic status and age was moderated the relationship -> age moderated the relationship

*Response 3*

*This has now been removed as the interaction is not statistically significant (L33-4).*

Comment 4

2) If there are significant interactions by age and sex,

In conclusions, authors concluded that green neighbourhoods reduce risk of CVD in older adults. However, because there was no association for those over 80 years of age and an inverse association for those at age -<80, I would suggest that authors delete "older adults".

*Response 4*

*We have now deleted ‘older adults’ (L36-8, L272-4).*

Comment 5

Please also include sex-difference in the abstract if interaction is significant.

*Response 5*

*The interaction between sex and greenspace is not significant.*

Comment 6

3) It is reasonable to think that neighbourhoods and communities are associated with behaviours. Validity is of higher priority than parsimony. Please include lack of information on diet, smoking, and alcohol intake as limitations in the discussion. If data are available, please state that inclusion of these variables did not alter the estimates and thus those were not included in the models.

*Response 6*

*Unfortunately, these data were not available for us to include in the model. We have now mentioned this as a limitation in the discussion (L325-6).*

Comment 7

4) For model 1 primary exposure and quantiles of greenspace, please specify whether it was quartile or quintile.

*Response 7*

*This was quartile, which we specify both in Table 3, the methods (L157) and the results (L219, 221), as well as in the tables. We are unsure as to where to provide further clarification.*

Comment 8

5) Page 16, line 250, Please replace SES (occupation) with occupation.

*Response 8*

*This has now been amended as suggested (L245). We have amended all references to social class where we are referring just to occupation in the manuscript (L249).*

Comment 9

6) in the discussion, does "actual" incident CVD mean that authors confirmed the cases through hospital records? Please clearly state that authors used hospital records rather than self-report. CVDs in other studies could be "actual" too, but their studies may also include false-positive cases.

*Response 9*

*Yes, actual incident CVD cases were confirmed through hospital records. This has now been clarified in the manuscript (L33, L279-81, Table 4 title).*
