# Supplementary material for: Residential neighbourhood greenspace is associated with reduced risk of cardiovascular disease: A prospective cohort study
Source: PLoS One. 2020 Jan 3;15(1):e0226524. doi: 10.1371/journal.pone.0226524 (PMC6941810; doi:10.1371/journal.pone.0226524)
Supplement: Attachment — Submitted filename: ResponseToReviewers.docx [file pone.0226524.s003.docx]

**Editor comments**

Comment 1

Journal Requirements:

When submitting your revision, we need you to address these additional requirements.

1. Please ensure that your manuscript meets PLOS ONE's style requirements, including those for file naming. The PLOS ONE style templates can be found at

http://www.journals.plos.org/plosone/s/file?id=wjVg/PLOSOne_formatting_sample_main_body.pdf and <http://www.journals.plos.org/plosone/s/file?id=ba62/PLOSOne_formatting_sample_title_authors_affiliations.pdf>

*Response 1*

*We have reviewed the style requirements. As a result, we have removed postcode information from the author affiliations and added the county.*

Comment 2

2. Thank you for submitting the above manuscript to PLOS ONE. During our internal evaluation of the manuscript, we still found significant text overlap between your submission and the following previously published works, on which you are an author.

https://doi.org/10.1186/s12889-016-3833-z

Please revise the manuscript to rephrase the duplicated text, cite your sources, and provide details as to how the current manuscript advances on previous work. Please note that further consideration is dependent on the submission of a manuscript that addresses these concerns about the overlap in text with published work.

We will carefully review your manuscript upon resubmission, so please ensure that your revision is thorough.

*Response 2*

*We have now further amended the text to remove overlap with our previously published study. We have checked these papers for duplication using Duplichecker.com which reports there is 0% duplicated content.*

Comment 3

3. We note that you have indicated that data from this study are available upon request. PLOS only allows data to be available upon request if there are legal or ethical restrictions on sharing data publicly. For information on unacceptable data access restrictions, please see http://journals.plos.org/plosone/s/data-availability#loc-unacceptable-data-access-restrictions.

In your revised cover letter, please address the following prompts:

a) If there are ethical or legal restrictions on sharing a de-identified data set, please explain them in detail (e.g., data contain potentially identifying or sensitive patient information) and who has imposed them (e.g., an ethics committee). Please also provide contact information for a data access committee, ethics committee, or other institutional body to which data requests may be sent.

b) If there are no restrictions, please upload the minimal anonymized data set necessary to replicate your study findings as either Supporting Information files or to a stable, public repository and provide us with the relevant URLs, DOIs, or accession numbers. Please see http://www.bmj.com/content/340/bmj.c181.long for guidelines on how to de-identify and prepare clinical data for publication. For a list of acceptable repositories, please see http://journals.plos.org/plosone/s/data-availability#loc-recommended-repositories.

We will update your Data Availability statement on your behalf to reflect the information you provide.

*Response 3*

*We have reviewed the above and address it in the cover letter under the heading ‘Study data’.*

Comment 4

4. Thank you for stating the following financial disclosure: "The funders had no role in study design, data collection and analysis, decision to publish, or preparation of the manuscript."

a.	Please provide an amended Funding Statement that declares *all* the funding or sources of support received during this specific study (whether external or internal to your organization) as detailed online in our guide for authors at http://journals.plos.org/plosone/s/submit-now.

b.	Please state what role the funders took in the study. If any authors received a salary from any of your funders, please state which authors and which funder. If the funders had no role, please state: "The funders had no role in study design, data collection and analysis, decision to publish, or preparation of the manuscript."

Please include your amended statements within your cover letter; we will change the online submission form on your behalf.

*Response 4*

*We include an amended statement within the cover letter under the heading ‘Funding Statement’.*

**Reviewer comments**

**Reviewer #1**

Comment 1

Thank you very much for giving me an opportunity to read this interesting manuscript. This study used information collected in Norfolk, UK and investigated the prospective association between residential neighborhood greenness and the risk of developing cardiovascular diseases. I have several questions and suggestions for the authors.

[Abstract]

- Please specify which year baseline information was collected (1993-1997?).

*Response 1*

*Baseline dates are now specified as 1993-1997 (L18-9).*

Comment 2

- (L.27) The authors stated the mean follow-up period was 17.8 years, which contradicts with the numbers I calculated from Table 2 (14-15 years).

*Response2*

*We apologise for this error. The correct figure is 14.5 years, which has now been amended throughout the manuscript (L27, L210, Table 3).*

Comment 3

- (L.30) The sentence “Physical activity did not mediate the relationship (HR = 0.99, 95%CI 0.97, 1.02; p = 0.475)” is not clear. What does this point estimate mean?

*Response 3*

*This refers to the HR attributable to the indirect effect physical activity (the mediator pathway), as opposed to the direct effect between greenspace and CVD, or the total effect considering both pathways. It compares the least green with the greenest quartile. The full result is given fully in Section 3.3. We now clarify this further in the abstract as ‘Physical activity did not mediate the relationship (****greenest compared to the least green quartile*** *HR 0.99; 95% CI 0.97, 1.01; p=0.416).’ (L30-1) and in the results as ‘A HR of 0.99 was attributed to the pathway through physical activity,* ***for the greenest compared to the least green quartile,*** *but this was not statistically significant (95% CI 0.97, 1.01; p=0.437).’ (L268-70).*

Comment 4

[Introduction]

- (L.49-65) The second paragraph is redundant. Needs to be revised.

*Response 4*

*We are unsure as to what the reviewers concerns are here. We feel this paragraph is important for setting out the state of the literature and current research gaps. We have updated the literature to include newer papers and papers highlighted during the review process (L50-65).*

Comment 5

- (L.57) What does “self-reported prevalent CVD”? Citation #10 used information collected in the UK Small Area Health Statistics Unit, which were all registered deaths.

*Response 5*

*The authors apologise for this, which is incorrect, and has now been deleted (now ref #11, L57).*

Comment 6

[Methods]

- Section numbers needs to be revised (2.1 → 2.1 → 2.2 → 2.1 → 2.2 → 2.3).

*Response 6*

*Revised.*

Comment 7

- (L.120-123) I disagree with their assumption. They only accounted for the period between 2000 and 2014 - but the participants had lived at the place for a certain amount of time already in 2000. In addition, for those who developed CVD and moved to another location, calculating the “equal time” for 2000 address and 2014 address means nothing. I recommend the authors just use baseline address for main analysis and conduct a sensitivity analysis in which they exclude those who moved after 2000. Their assumption is theoretically and statistically irrelevant.

*Response 7*

*We have now amended the analysis to measure exposure only according to green space in the neighbourhood in the year 2000 (L113+). We note that this was the approach we took in the original submission of this manuscript, but we amended it to a measure of average green space based on earlier reviewer recommendation. We conducted a sensitivity analysis in which all people known to have who moved after 2000 and those who may have moved (for whom we had no postcode information after 2000) were excluded. Strength of effect, direction of association and statistical significance were unaffected. These results have now been added to the manuscript (L278-83).*

Comment 8

- (L.126) Please provide more information on how PA was estimated (e.g., information on the questionnaire used, cut-offs to create categories...).

*Response 8*

*We now explicitly reference the questionnaire (in previously cited reference #43) and cut-offs used to create our chosen categories (now in Table 2) (L132+).*

Comment 9

- (L.153) How did you define the last date of observation period for those who were lost to follow-up?

*Response 9*

*Hospital records were used up to 31/03/2016, so for people who were lost to follow-up, this date represents the last observation period as we were still able to obtain data on incident CVD. We now add in the following sentence to clarify this: ‘If participants were lost to follow-up, CVD outcome data could still be accessed using hospital records’. (L166-7).*

Comment 10

[Results]

- (L.180-185) These sentences should appear in Method section.

*Response 10*

*Information on excluded participants has been moved to the Method section (L153-9).*

Comment 11

- (L.193) Please information on the numbers of [1] fatal CVD cases, [2] non-fatal cases, [3] those who died due to causes other than CVD, [4] those who were lost to follow-up (censored cases) and [5] those who were alive at the end of observation period.

*Response 11*

*The figures are as follows:*

*[1] 5734 (564 of these died after the date of CVD, but we do not have the cause of death), [2] 7545, [3] 1829, [4] 3 people opted out of the study so were not included in analysis. For the other participants, hospital linkage means that even if they did not take part in follow-up surveys, we were able to access outcome data on CVD incidents [5] 16857. These figures have been added into the manuscript where relevant (L206-10).*

Comment 12

- (L.196) Please check if the follow-up period is correct (17.8 years).

*Response 12*

*Apologies; this should be 14.5 (see response 2).*

Comment 13

- (L.198) Why presented three types of PA results? I also don’t understand why they did not provide detailed information (hrs per week) for the last PA result.

*Response 13*

*We have now simplified this. We present the four level overall physical activity measure, along with categories of recreational activity, and total hours walked per week in the summer (Table 3, L211).*

Comment 14

- (L.236) Please clarify that models were adjusted for both individual- and neighborhood-level SES.

*Response 14*

*Clarified (L257, L276, L304).*

Comment 15

- Please provide results of proportional hazard assumption (e.g., Schoenfeld Residuals Test)

*Response 15*

*We now present the results of the Schoenfeld Residuals Test as text in the manuscript (L239-41), which showed potential violation of the model for four variables. We carried out piecewise analysis to further investigate this, by exploring changes in hazard ratios of these time-varying covariates (L242-8). To overcome potential model violation, we added interaction terms for each of the time-varying covariates into the adjusted model. Three of these were statistically significant, and their inclusion showed a statistically significant improvement in model fit (by p<0.001), but had little effect on exposure effect or statistical significance. We have added these additional results into the manuscript (L248-53).*

Comment 16

- (L.242) Please provide a table for mediation analysis.

*Response 16*

*Tables for the mediation analysis have now been added in for the main results (Table 5, L273) and for the sensitivity analysis in the Supplementary table S1.*

Comment 17

[Discussion]

- (L.288-290) The biogenics hypothesis does not seem to be accepted as much as the other interpretations. I recommend the authors should delete (or at least tone down) the sentence.

*Response 17*

*This sentence has now been revised down by indicating that there is less evidence to support the biogenesis hypothesis, yet it still has not been disproved (L333-5).*

Comment 18

- (L.308-309) Please try the sensitivity analysis mentioned above.

*Response 18*

*This has been carried out and the discussion has been amended (L353-6).*

Comment 19

- Lack of information on the access to gym or swimming pool can be a limitation of this study. These places have nothing to do with greenness but provide an opportunity for the residents to engage in physical activities.

*Response 19*

*The physical activity variable included activities such as swimming and the gym. In the questionnaire, they were asked “In a typical week during the past 12 months, how many hours did you spend on each of the following activities? 1. Cycling 2. Other physical exercise such as keep fit, aerobics, swimming, jogging”. We now explicitly reference the questionnaire, and have added in these activities into the methods section (L132+). In terms of our exposure measure, we were investigating the role of greenspace specifically, rather than sporting venues or other locations for physical activity.*

Comment 20

- I think the authors should emphasize that they observed the association between neighborhood greenness and incident CVD even after adjusting for neighborhood SES. Some might suspect that rich neighborhoods have a better environment (e.g., more parks) and that greenspace is just an confounder in the association between neighborhood SES and CVD, particularly because the authors did not show any evidence of mediation by physical activity.

*Response 20*

*This has now been emphasised in the Abstract (conclusions) (L37-8) and the Conclusions (L379-80).*

**Reviewer #2**

Comment 21

This study adds to the building literature on greenspace and cardiovascular disease by modeling time-dependent outcomes in a population-based cohort of Britons from the county of Norfolk. Another interesting component of this manuscript is its analyses of effect mediation & moderation, establishing that most of the beneficial outcomes associated with nature exposure do not seem to be attributable to physical activity, and that there are interesting relationships among specific subpopulations defined according to occupation.

How granular are postcodes in the UK? In the US, researchers commonly consider census block group (600-3000 population) to be a suitable level of geographic resolution for characterizing neighborhood effects. Zip-code level characterizations are far more crude and can smooth over important geographic differences in exposures. Generally speaking, the result of using too large of areas for spatial epidemiologic studies is that it tends to bias results toward the null. The sensitivity analysis of different definitions of neighborhood exposure seem to address this issue, although it probably should be stated how many residents tend to live within a given postcode in the UK and additionally the description of the buffer-based neighborhood definitions needs to be made more specific (e.g., were these buffers defined in reference to individuals addresses of residence? What is a "road" buffer type?).

*Response 21*

*Postcodes in the UK cover smaller areas than US zip codes. We have now clarified this in the manuscript (L118-21), stating “Each of the 1.3 million postcodes in England and Wales covers on average 42.8 people (min 1, max 3215; st dev 38.8) and 17.9 households (min 0, max 646; st dev 14.8). These cover a smaller area than the smallest Census unit (output area) of which there are 175,434 in England and Wales. Regarding neighbourhood definitions, we had stated that the neighbourhood was based on home postcodes, but we now make this more explicit. A road buffer uses the road network to calculate the area that can be accessed by an individual travelling a certain distance along the roads, rather than just using a straight-line distance from the postcode. We now clarify this in the methods (L125-7).*

Comment 22

Cardiovascular risk is complex and is affected by physiological, hereditary, behavioral factors in addition to environmental characteristics. The analysis accounted for age, sex, BMI, diabetes and socioeconomic status. At a minimum, adjustment for generally-accepted clinical risk factors for cardiovascular outcomes (e.g., ACC/AHA Pooled Cohort Equations risk factors) would be recommended in order to better address potential confounding.

*Response 22*

*We only adjust the models for potential confounding factors ie factors that confound the relationship and are thereby associated with both exposure (green space) and outcome (CVD). As is standard for this form of analysis, risk factors for CVD that are not also hypothesised to be associated with greenspace exposure are therefore not included.*

Comment 23

Housing costs would seem to be another potential confounding variable. Within urbanized areas, housing costs negatively correlate with greenspace exposure. How are the authors thinking about housing in general, aside from percent renting and overcrowing as reflected in the Townsend Index?

*Response 23*

*Housing costs are not included in the model as they are highly correlated with greenspace and with the Townsend Index, a measure of neighbourhood SES, to account for neighbourhood wealth/deprivation. We do not believe housing costs, which are a relatively poorly measured variable in the UK, would add strong predicative power to the model over and above the impact of the Townsend Index. We have added this into the limitations section of the discussion (L365-8).*

Comment 24

The fact that physical activity did not mediate the observed relationship between greenspace exposure and CVD incidents is interesting, and coheres with other work on greenspace exposure (e.g. Maas et al., J Epi & Comm Hlth 63.12 967-973, which I see the authors have cited).

*Response 24*

*We thank the reviewer for highlighting this. We have now referred to the literature in the discussion (L321-3) to highlight that our findings align with other work (noting that the Maas et al paper only hypothesises physical activity as a mediator rather than explicitly testing for it).*

Comment 25

I would have liked to have seen more details on the moderation analysis results, perhaps in the form of a table or supplemental table. I couldn't follow the part of Section 3.3 that summarized the results of that analysis. Furthermore, what are some hypotheses about why individuals in certain social contexts (e.g., occupations) may demonstrate stronger/weaker associations between green space exposure and CVD events?

*Response 25*

*We chose not to present data for interaction effects that were non-significant, as we did not feel that this contributed to the paper. We have now simplified these results (L284-95), and provide further data/clarification, which should make the section easier to follow. We hypothesise that occupation may be relevant in this context, due to the present of confounding factors not tested in this analysis. For example, it could be that people in non-manual occupations are more likely to work in an office rather than outdoors, therefore living near greenspace may matter more to them. We now add this into the discussion (L313-56).*

Comment 26

Living near greenspace is one mode of exposure to nature (Frumkin et al., Envir Hlth Perspectives, 2017). Residing in a green neighborhood, as characterized by these authors, represents a routine exposure to nature at an intermediate spatial scale. Other studies have indicated prolonged effects of more intense, short-term exposures to nature, such as improved natural killer cell activity (PMID: 21329564). I guess my point is that there are several ways we can think about exposure to nature, and that these may have specific and/or shared mechanisms that support improved CVD risk profiles. The authors state that the causes of the relationship between greenspace exposure and CVD are largely unexplained, and while I don't disagree with that statement, there is a growing body work in this field (as highlighted well by the Frumkin paper).

*Response 26*

*Thank you for drawing our attention to this paper. We have now added this, as well as some additional literature, to our discussion (L328-335).*

Comment 27

On a related note, residential location data may not represent activity spaces of individuals. The authors mention the limitation of not having record of moves that occurred between 2000 and 2014, but this would be an additional complicating aspect of characterizing individuals' exposure to nature.

*Response 27*

*We have amended the discussion to state ‘Use of greenspace amongst our participants was not known, we do not know if residential locations represent activity spaces of individuals, and we did not use a measure of publicly accessible greenspace. However, although it may be that just the presence of any greenspace may benefit health.’ (L357-60)*

Comment 28

The fact that physical activity did not mediate the observed relationship between greenspace exposure and CVD incidents is interesting, and coheres with other work on greenspace exposure as discussed in the above comment.

*Response 28*

*See response 24, above.*

Comment 29

Were there steps taken to ensure the replicability of the study (e.g., data/code archiving, automated/reproducible report generation)?

*Response 29*

*All data is available via a data sharing agreement with the EPIC-Norfolk management team. Data/code archiving and automated/reproducible reports were not generated.*

Comment 30

Other comments:

The definition of the greenspace exposure that was given in the Abstract was very difficult to comprehend.

*Response 30*

*We have amended the definition to ‘Greenspace exposure was identified using classified satellite imagery.’ (L20-1).*

Comment 31

Likewise, the statement of the main result (7% lower relative hazard for those living in the greenest neighborhood quartile) did not specific a referent neighborhood greenspace exposure group or value.

*Response 31*

*The comparison group is now clarified (L28, L30, L234, L269-70).*
